# Modulating Thermal Conductivity and Flame Retardancy of Polyolefin Composites via Distributed Structures of Magnesium Hydroxide and Hexagonal Boron Nitride

**DOI:** 10.3390/polym16050646

**Published:** 2024-02-28

**Authors:** Qi Wang, Lin Pan, Ruitian Bo, Yunfei Wang, Zhidong Han

**Affiliations:** 1 School of Materials Science and Chemical Engineering, Harbin University of Science and Technology, Harbin 150080, China; hlgwangqi0514@163.com (Q.W.);; 2 Key Laboratory of Engineering Dielectrics and Its Application, Ministry of Education, Harbin University of Science and Technology, Harbin 150080, China

**Keywords:** polyolefin composites, magnesium hydroxide, hexagonal boron nitride, flame retardancy, thermal conductivity

## Abstract

Thermally conductive and flame-retardant polyolefin composites are facing great challenges in meeting the increasing demands for fire safety and thermal management. Aiming at simultaneously enhancing thermal conductivity and flame retardancy, hexagonal boron nitride (hBN) and magnesium hydroxide (MH) were adopted in ethylene–vinyl acetate copolymer/polyolefin elastomer (EVA/POE) blends to design composites with selective filler distributions and co-continuous networks via different processing schemes. The thermal conductivity and flame retardancy show strong dependence on the distributed structure of hBN and MH. The composites with hBN-rich centers and MH-rich edges in the filled POE phase show a thermal conductivity of 0.70 W/(m·K) and an LOI of 27.7%, which are very close to the thermal conductivity of EVA/POE/hBN and the LOI of EVA/POE/MH at the same total filler content. The composites with MH-rich centers and hBN-rich edges show pHRR, THR and TSP values of 169 kW/m^2^, 49.8 MJ/m^2^ and 1.8 m^2^, which are decreased by 40%, 33% and 62% in comparison with EVA/POE/MH, respectively. Modulating the filler structure distribution provides a strategy to co-enhance thermal conductivity and flame retardancy.

## 1. Introduction

Polyolefins are the most widely applied plastic materials, due to their characteristic performance benefits such as being lightweight, as well as their chemical inertness, insulation properties and structural tunability [1,2]. With the rapid progress in the field of new materials technology, polyolefins are facing enormous challenges to meet the increasing demands for fire safety, thermal management and dielectric performances. In this instance, their inherent flammability and low thermal conductivity are the main issues to fire hazards and thermal damage when polyolefin materials are used in the fields of electrical equipment and electronic components. Taking insulation and sheath materials in wires and cables as an example, the requirements for the thermal conductivity and flame retardancy of polyolefin composites are becoming increasingly prominent in high-power operation and high-density layout situations [3]. Thus, as a widely used strategy, inorganic additives are introduced to enhance the flame retardancy and thermal conductivity of polyolefin composites. For efficient heat transfer and fire safety, it is of great practical significance to explore polyolefin materials with both thermal conductivity and flame-retardant properties.

Currently, varieties of halogen-free inorganic flame retardants are used in polyolefins, including metal hydroxides, metal hydroxide carbonates and phosphorus-containing inorganic flame retardants [4,5]. Among them, magnesium hydroxide (MH) draws great attention because of its non-toxic, low smoke and environmentally friendly characteristics. MH exerts its flame retardant influence through dehydration and heat absorption in its condensed phase, and its action inevitably yields disadvantages of low flame retardant efficiency and large loading levels (more than 60 wt.%) [6]. Much effort has been devoted to enhance the flame retardancy of MH in polyolefins with synergistic additives, including polycarbosilane as a ceramic agent in ethylene-vinyl acetate copolymer (EVA) [7], lanthanum oxide as a catalytic synergist in polypropylene (PP) [8], montmorillonite as a nanosized filler in polyethylene (PE) [9], and so on. Significant progress has been made in the field of MH flame-retardant polyolefins; however, few studies have been delivered on the application of MH in polyolefin composites co-enhanced with thermal conductivity.

For thermally conductive composites of polyolefins, hexagonal boron nitride (h-BN) is considered as an ideal filler, thanks to its high thermal conductivity and electrical insulation [10]. Layer-structured h-BN is held together by van der Waals forces, and a single layer is typically referred to as a BN nanosheet (BNNS). The enhanced thermal conductivity of the composites is influenced by their BN content, particle size and morphology, surface modification, compatibility with matrixes and synergistic effects with other fillers [11]. In order to achieve thermally conductive composites, a distributed structure of h-BN was constructed to form an efficient path for heat transfer. The segregated structure of h-BN was built to endow ultrahigh molecular weight polyethylene (UHMWPE) with a thermal conductivity of 9.99 W/(m·K) [12]. Thermally conductive pathways of BNNSs and nanospheres (BNNOS) were constructed to obtain modified PP composites with a thermal conductivity of 0.514 W/(m·K) [13]. BNNS-laminated structures were also reported to show a superior in-plane thermal conductivity of 13.2 W/(m·K) for EVA composite films [14].

BN has also found applications in flame-retardant composites [15,16,17]. In recent research from Yao et al. [18], polyphosphazene-modified MH crosslinked by boric acid was reported to grow BN in situ, which hindered the transfer of volatile products and formed a compact char layer in the condensed phase of EVA. Some attempts have recently been made to take into account the flame retardancy and thermal conductivity of the composites. Multifunctional nanofillers based on BNNSs and biobased phytic acid (f-BNNSs) showed a thermal conductivity enhancement of 261% and an LOI enhancement of 49% on poly (L-lactic acid) (PLLA) at a loading of 20 wt.% [19]. Modified BN with vinyltrimethoxysilane and 9,10-dihydro-9-oxa-10-phosphaphenanthrene-10-oxide showed synergistic effects with aluminum diethyl phosphinate in styrene–butadiene rubber (SBR), and the thermal conductivity of the composites was improved to a certain extent [20].

The characteristics of both BN and MH are their non-toxicity, environmental friendliness and good thermal stability [21,22]. MH is normally thermally stable up to 330–340 °C, and h-BN is stable at temperatures of over 1000 °C in air. Therefore, they can be used in most types of thermoplastics, thanks to their wide product categories in terms of particle size and morphology. However, MH and BN have rarely been applied together in polyolefin composites to co-enhance their flame retardancy and thermal conductivity, because both MH and BN show performance that depends on their loading levels. It would a big challenge for polyolefin composites to simultaneously achieve good flame-retardant properties and good thermally conductive performance while keeping their filler loading at moderate levels. In view of this issue, the distributed structure of MH and BN was investigated in EVA and polyolefin elastomer (POE) to construct flame-retardant and thermally conductive polyolefin composites. The strategy would provide a feasible route to meet the comprehensive requirements of polyolefin composites in manufacturing scenarios for practical applications such as wires and cables.

## 2. Materials and Methods

### 2.1. Materials

The ethylene–vinyl acetate copolymer (EVA, 7470M) came from Ningbo Formosa Plastics Co. (Ningbo, China). The thermoplastic polyolefin elastomer (POE, DF740) came from Mitsui Chemicals Inc. (Tokyo, Japan). The magnesium hydroxide (MH, KISUMA^®^5-C) was from KISUMA (Dandong) High-tech Material Technology Co. (Dandong, China). Hexagonal boron nitride (hBN) with a particle size of 1 µm was acquired from Shanghai Aladdin Biochemical Technology Co. (Shanghai, China), and BN is adopted as its abbreviation henceforth.

### 2.2. Preparation of the Composites

**Figure 1:**
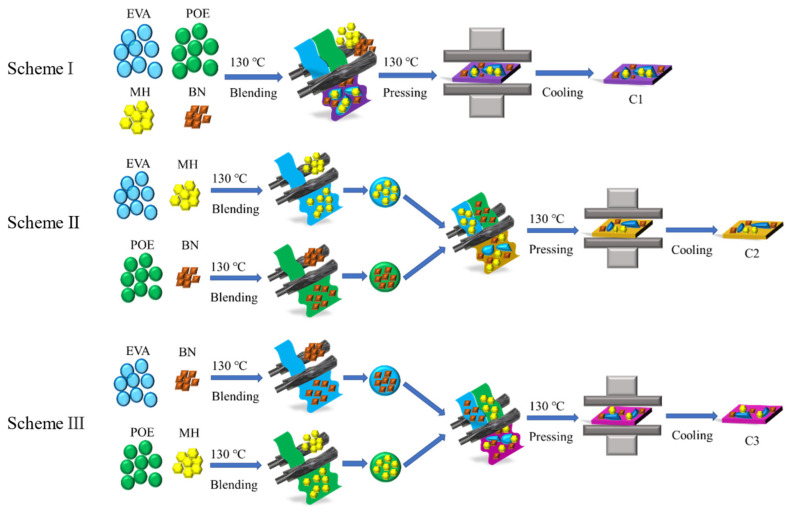
Processing scheme of the composites.

The composites were designed according to an EVA/POE mass ratio of 1/1, an MH/BN mass ratio of 1/1, and a matrices/fillers mass ratio of 1/1, which resulted in a composition of 25 wt.% EVA, 25 wt.% POE, 25 wt.% MH and 25 wt.% BN, as shown in Table S1. The composites were prepared by a melt blending method with a torque rheometer (Changchun Smart Control Technology Co., Ltd., Changchun, China). The processing was performed at a temperature of 130 °C and a rotating speed of 30 rpm. Three processing schemes were designed to prepare the composites with different filler distributions, as shown in Figure 1.

For Scheme I, all of the materials were added into the rheometer and blended for 10 min to obtain the composites of EVA/POE/MH/BN, named as C1. For Scheme II, the composites of EVA/MH and POE/BN were prepared by blending in the rheometer for 10 min, and then the two kinds of EVA/MH and POE/BN composites were blended together in the rheometer for 10 min at a mass ratio of 1/1 to obtain the composites of (EVA/MH)/(POE/BN), named as C2. For Scheme III, the composites of EVA/BN and POE/MH were prepared by blending in the rheometer for 10 min, and then the two kinds of EVA/BN and POE/MH composites were blended together in the rheometer for 10 min at a mass ratio of 1/1 to obtain the composites of (EVA/BN)/(POE/MH), named as C3. The samples and the processing procedure are shown in Table S2. All of the samples were pressed with a flatbed vulcanizing machine (Shanghai Jiubin Instruments Co., Shanghai, China) at 130 °C and 10 MPa for 15 min.

### 2.3. Characterization

Selective solvent extraction experiment was performed to reveal the co-continuous morphological structure of the composites. The composite sample was immersed in ethyl acetate solution at 60 °C for 5 h, and then the sample was removed from the solution and washed 3 times with ethyl acetate and ethanol.

A scanning electron microscope (SEM, Apreo, FEI, Hillsboro, OR, USA) was used at an operating voltage of 20 kV. The composite samples were cryo-fractured after immersion in liquid nitrogen. Fractured sections were metallized with gold to observe the morphology of the composite. The surface of the residual char after CONE testing was also metallized to observe the morphology of the char. The structure of the char after CONE testing was characterized using an X-ray diffractometer (XRD, D/maxr B, Rigaku Electric, Tokyo, Japan) with Cu Kα. The analysis was performed at a step size of 0.026 nm, a tube voltage of 40 kV, a tube current of 30 mA, a nickel chip filter and a scanning rate of 8°/min.

The limiting oxygen index (LOI) was tested with an oxygen index tester (JF-3, Nanjing Jiangning Analytical Instrument Factory, Nanjing, China) according to ASTM D2863 [23], with a specimen that was 100 mm long, 6.5 mm wide and 3.0 mm thick. Vertical burning was tested using a tester (CZF-3, Nanjing Jiangning Analytical Instrument Factory, Nanjing, China) according to the UL 94 standard [24], with a sample that was 100 mm long, 13 mm wide and 3.0 mm thick. The combustion behaviors were evaluated according to ISO 5660 [25] on a CONE calorimeter at a heat flux of 35 kW/m^2^ with square specimens 100 mm long, 100 mm wide and 3.0 mm thick.

The thermal diffusivity (*α*) of samples with a diameter of 12.6 mm and a thickness of 1 mm was tested at 25 °C using a laser flash analyzer (LFA447, Netzsch, Weimar, Germany). The samples were sprayed with a graphite coating prior to testing. The thermal conductivity of the composites can be calculated according to the equation of *λ* = *α·ρ·C_p_*, where *ρ* and *C_p_* represent the density and specific heat capacity, respectively. The temperature profile on the upper surface of composite samples with a diameter of 12.5 mm and a thickness of 1.0 mm was monitored using an infrared thermal imager (FOTRIC-220S, FOTRIC INC., Shanghai, China).

## 3. Results

### 3.1. Distribution of BN and MH in EVA/POE Blend

**Figure 2:**
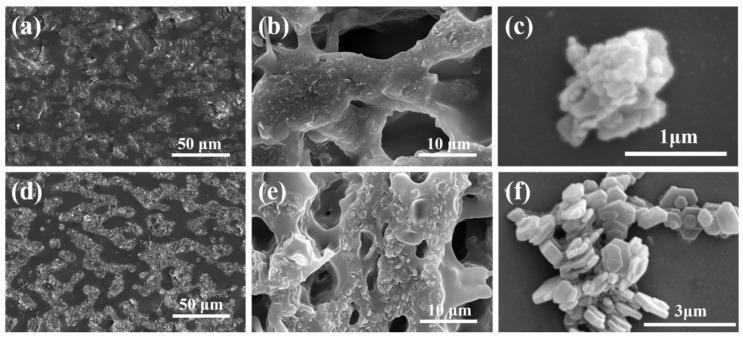
SEM images of the composites of EVA/POE/BN (**a**) and EVA/POE/MH (**d**); extracted composites of EVA/POE/BN (**b**) and EVA/POE/MH (**e**); the fillers of BN (**c**) and MH (**f**).

BN and MH were dispersed in the blend of EVA/POE to obtain the EVA/POE/BN and EVA/POE/MH composites. Figure 2 shows SEM images of the composites and those after selective extraction, together with images of the BN and MH particles. As observed in Figure 2a,d, the composites show a two-phase structure, one phase with filler particles and the other without filler particles. Such phenomena reveal the selective distribution of BN and MH particles in the EVA/POE blend. Selective extraction was carried out on the composites to dissolve the EVA component, utilizing the solubility differences of EVA and POE in ethyl acetate. As shown in Figure 2b,e, the holes are left due to the removal of the EVA component, and the POE component is left as residues due to its insolubility in ethyl acetate. Meanwhile, the dispersed particles are found in the residues, and the extracted solutions are clear without filler particles. Compared with the particle images in Figure 2c,f, it can be inferred that BN and MH are distributed in the POE phase of the blend. Accordingly, the EVA phase and the POE/BN phase form a co-continuous network in the EVA/POE/BN composites, while the EVA phase and the POE/MH phase form a co-continuous network in the EVA/POE/MH composites.

### 3.2. Distributed Structure under Processing Scheme

**Figure 3:**
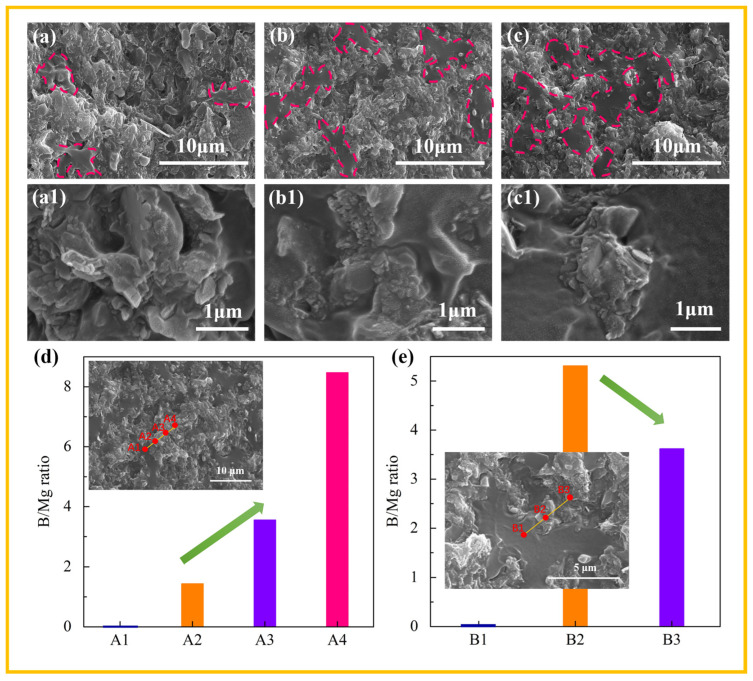
SEM images with different magnifications of C1 (**a**,**a1**), C2 (**b**,**b1**) and C3 (**c**,**c1**). The B/Mg ratios at the different positions of C2 (**d**) and C3 (**e**) in the inserted SEM images.

BN and MH were added together into the EVA/POE blend to endow the composites with thermal conductivity and flame-retardant properties. The distributed structure of the fillers was modulated by different processing schemes in Figure 1, and the SEM images of the composites are shown in Figure 3. The two-phase structure of the composites can be observed. According to the selective distribution of both BN and MH in the POE, the mixed fillers of BN and MH are found in the POE, and the phase structure forms with the EVA phase and the filled phase of the POE/MH/BN. Note that there are significant differences in the dispersed dimensions of the EVA phase in the composites from different processing schemes, as indicated by the lines in Figure 3. The EVA phase dimension shows an order of C1 < C2 < C3. When observed at the higher magnifications, different distributed structures of BN and MH are found in the composites. The fillers in C1 are randomly distributed in the filled POE phase, while some segregated distributions of BN and MH are revealed in C2 and C3. The larger-sized particles of MH surround smaller particles of BN in C2, and the BN particles center around the MH particles in C3.

To specify the structural characteristics of C2 and C3, the EDS spectra shown in Figure S1 were collected at different positions along the line from the EVA phase to the filled POE phase. The elemental ratios of B and Mg were calculated to indicate the distribution structure of BN and MH, as shown in Figure 3d,e. No peak of element B is present in the EDS spectra at the positions of A1 for C2 and B1 for C3. Since the positions of A1 and B1 are located in the EVA phase, this proves the selective distribution of BN in POE. As the position goes from the edge to the center of the filled POE phase, profoundly different B/Mg ratios are revealed. For C2, the B/Mg ratio increases dramatically, and the BN-rich central part is accordingly proved, which evidences the structure of MH surrounding BN in Figure 3(b1). Instead, the B/Mg ratio decreases dramatically for C3, proving the MH-rich central part, which evidences the structure of BN centering around MH in Figure 3(c1).

Accordingly, the distributed structure of BN and MH can be designed and achieved by different processing schemes. The BN-rich center forms in C2 by Scheme II, and the MH-rich center forms in C3 by Scheme III. During processing, the selective distribution behaviors of BN and MH in the EVA/POE blend drive filler migration and contribute to the formation of MH-rich edges in C2 and BN-rich edges in C3. Thus, the noticeable structured composites are obtained by building the segregated structure of BN and MH in C2 and C3 with the processing schemes. The segregated distribution of BN and MH influences the thermal conductivity and flame-retardant properties of the composites.

### 3.3. Thermal Conductivity of the Composites

**Figure 4:**
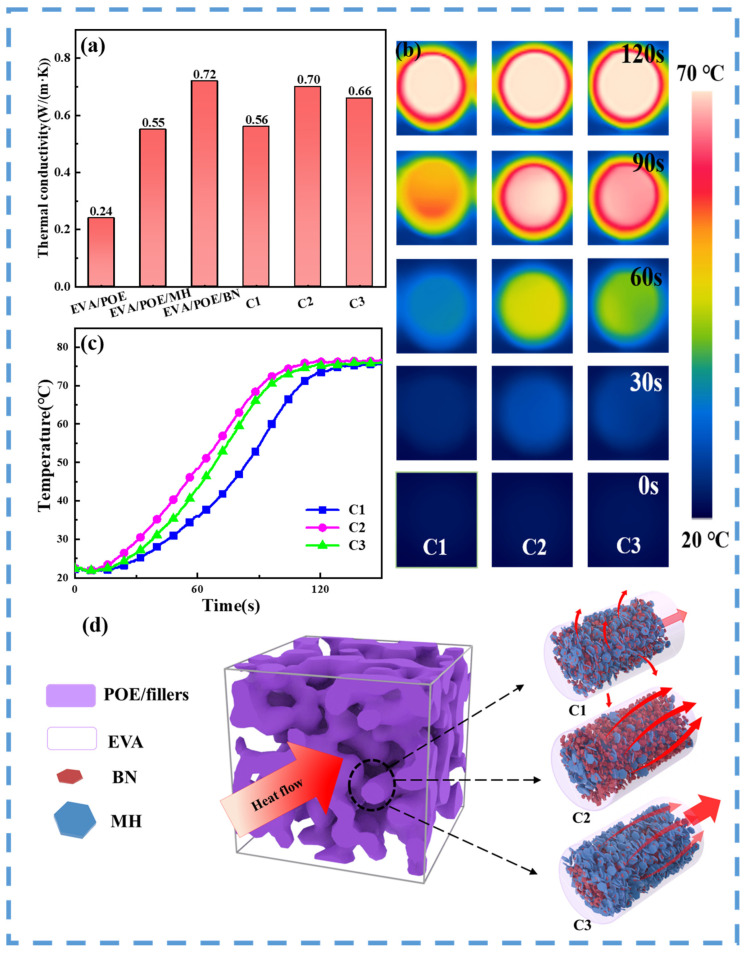
Thermal conductivities of the composites (**a**), infrared thermographic diagram (**b**), surface temperature vs. time curves (**c**) and the schematic diagram of the distributed structure (**d**).

The thermal conductivities of the composites prepared with combined BN and MH via different processing schemes were compared with those with the single kind of fillers in Figure 4a. EVA/POE at a mass ratio of 1/1 shows a low thermal conductivity of 0.24 W/(m·K). With a filler content of 50 wt.%, EVA/POE/MH and EVA/POE/BN present thermal conductivities of 0.55 W/(m·K) and 0.72 W/(m·K), respectively. In comparison with the EVA/POE, the thermal conductivities are increased by 129% and 200%, respectively. BN as a thermally conductive filler shows more impressive effects than MH in enhancing the thermal conductivity of the composites. As a result, EVA/POE/BN shows a 31% increase in thermal conductivity in comparison with EVA/POE/MH. For the composites filled with both BN and MH at a mass ratio of 1/1, the processing schemes significantly impact the thermal conductivity of the composites. C1 shows a thermal conductivity of 0.56 W/(m·K), which is similar to EVA/POE/MH. C2 and C3 show thermal conductivities of 0.70 W/(m·K) and 0.66 W/(m·K), respectively, which are similar to EVA/POE/BN.

In viewing of morphology of the composites prepared by different processing schemes, the remarkable disparities in the thermal conductivity arise from the distributed structure of BN and MH. For C1, the randomly distributed BN and MH bring resistance for BN to build a path for heat transfer, which leads to the inferior thermal conductivity. Even though the mixed filler contains half a percent of BN, C1 shows a thermal conductivity that is similar to EVA/POE/MH, and fails to take advantage of the high thermal conduction efficiency of BN. For C2 and C3, the segregated structure of BN and MH forms by the migration of fillers from the EVA phase to the filled POE phase during processing. The BN-rich center in the filled POE phase of C2 and the BN-rich edge in the filled POE phase of C3 can facilitate the formation of an efficient BN path for heat transfer and high thermal conductivity. Although their content of BN is reduced by half, C2 and C3 present thermal conductivities similar to EVA/POE/BN, and fully utilize the function of BN. Compared with C3, C2 presents higher thermal conductivity with its segregated BN center in the heat path.

The heat transfer capabilities of composites from the three processing schemes can be visually reflected by infrared thermography testing. Figure 4b illustrates the color diagrams of the composites during infrared thermography testing, and Figure 4c shows the curves of temperature vs. heating time. According to the sample color during the heating procedure, the composites show an order of heat transfer capability that is C2 > C3 > C1, which shows good consistency with the thermal conductivity results. The higher temperature for the same heating time in Figure 4c provides further evidence for the better heat transfer capabilities of C2 and C3 than that of C1. The difference in the heat transfer capability can also confirm the effects of the filler distribution and the superiority of a segregated structure in enhancing thermal conductivity and heat transfer. Consequently, the thermal conductivity of the composites shows strong dependence on the fillers and their distributions. As illustrated in Figure 4d, the segregated BN distribution facilitates heat transfer and enhances thermal conductivity at a lower BN content. As evidenced, C2 has a similar thermal conductivity to EVA/POE/BN, despite of the halved BN content.

### 3.4. Flame-Retardant Properties of the Composites

**Figure 5:**
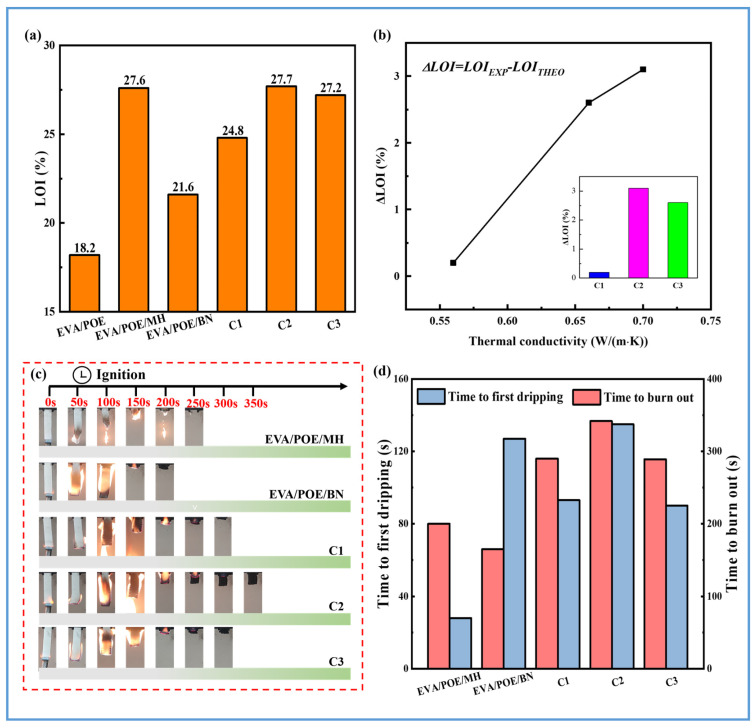
The LOI values of the composites (**a**) and the curve of ΔLOI vs. thermal conductivity (**b**); the photographs during UL-94 testing (**c**) and the time parameters during the burning (**d**).

The LOI results of the composites are illustrated in Figure 5a. The EVA/POE shows an LOI of 18.2%, while the EVA/POE/BN and EVA/POE/MH show LOIs of 21.6% and 27.6%, respectively. It can be seen that the LOI enhancement of the composite with BN is very limited in comparison with that with MH. The EVA/POE/MH shows a 28% increase in LOI over the EVA/POE/BN. By filling both BN and MH in the EVA/POE, the composites from the three processing schemes present enhanced LOI values over the EVA/POE/BN. The processing scheme exerts a strong influence on the LOIs of the composites. C1 shows an LOI of 24.8%, which is higher than EVA/POE/BN and lower than EVA/POE/MH. C2 and C3 show LOIs of 27.7 and 27.2, respectively, which are close to that of EVA/POE/MH. Compared with C1, the significant LOI enhancement of C2 and C3 is revealed for the same content of BN and MH.

When the content of BN and MH is considered, theoretically calculated LOI values (*LOI_THEO_*) can be obtained for the composites with formulations of EVA/POE/MH/BN according to the LOI values of EVA/POE/BN and EVA/POE/MH. Then, the difference value (ΔLOI) is calculated by the experimental LOI values (*LOI_EXP_*) minus *LOI_THEO_*. ΔLOI can be used as a parameter to indicate the synergistic effect between BN and MH. As shown in Figure 5b, the ΔLOI value of C1 is 0.2%, indicating a weak synergistic effect between BN and MH in C1. For C2 and C3, the high ΔLOI values of 3.1% and 2.6%, respectively, reveal a strong positive synergistic effect between BN and MH in C2 and C3. The different ΔLOI results can be attributed to the distribution structure of BN and MH. The randomly distributed structure of BN and MH in the POE phase may be a reason for the weak synergistic effect, while the segregation distribution of BN and MH may contribute to the synergistic effect between BN and MH. Furthermore, C2 illustrates a higher LOI than C3, indicating the segregated structure with a BN-rich center and MH-rich edge in the filled POE phase would be favorable for flame retardancy. Since the composites from the three processing schemes are composed of the same formulations, the flame retardancy of the composites shows a relationship with thermal conductivity, as illustrated in Figure 5b. The high thermal conductivity reflects the high LOI when the composition of the composites is the same.

Figure 5c shows digital photographs from the vertical burning test. The parameters during the vertical burning test are listed in Table S3. Note that all of the samples burned out after the first ignition, with no rating achieved. In this scenario, the time to burn out was adopted as a parameter to evaluate the burning behavior. Taking dripping phenomena into account, the time to first dripping was also recorded. According to the time to burn out and the time to drip shown in Figure 5d, the EVA/POE/BN took less time to burn out and longer to drip, which can be related with its lower LOI and higher thermal conductivity. Compared with the EVA/POE/MH, the composites filled with BN and MH show longer times to burn out and drip, indicating the synergistic effect between BN and MH during the burning test. Among the composites from the three processing schemes, C2 shows the longest time to burn out and drip, which is in correspondence with its high LOI and high thermal conductivity. The factors that affect burning behaviors are complex, which are not only linked to the flame retardancy of the composites, but also to the heat released during burning, the thermal conductivity of the composites and the residual char. Considering the flame retardancy and thermal conductivity of the composites, the higher thermal conductivity leads to drip for a longer time after ignition, while the higher LOI makes for the delayed burning rate and the prolonged burning procedure. C2 takes advantage of both factors, thanks to the segregated structure of BN and MH, and modulates the burning behaviors by showing the first dripping at 135 s and flame out at 352 s, which are delays of 97 s and 142 s, respectively, in comparison with the EVA/POE/MH.

### 3.5. Combustion Behaviors of the Composites

**Figure 6:**
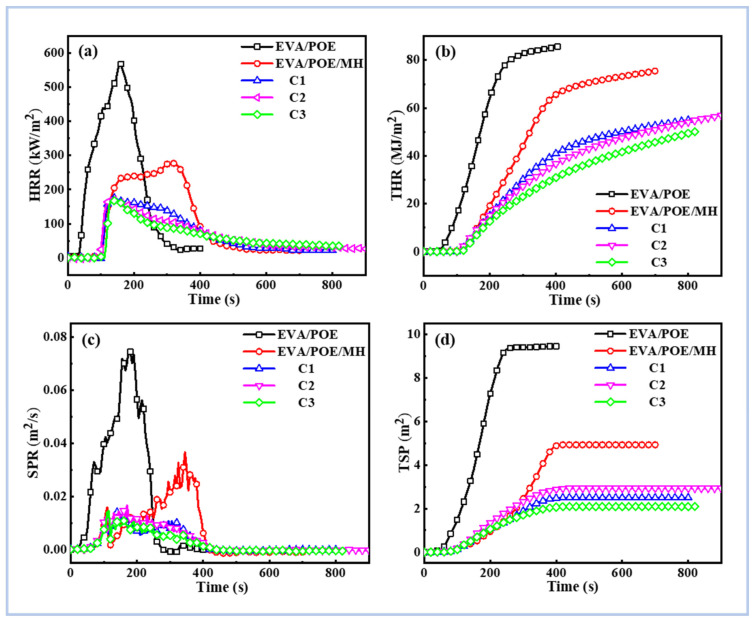
The combustion behaviors of HRR (**a**), THR (**b**), SPR (**c**) and TSP (**d**) of the composites via CONE calorimetry.

The combustion behaviors of the composites were evaluated using a CONE calorimeter, and the results are shown in Figure 6 and Table S4. For the peak heat release rate (pHRR) and total heat release (THR), the EVA/POE/MH shows pHRR and THR values of 281 kW/m^2^ and 74.8 MJ/m^2^, which are 37% and 13% lower than the EVA/POE, respectively. The pHRR and THR of C1 are further reduced to 178 kW/m^2^ and 54.7 MJ/m^2^, which are 60% and 36% lower compared to the EVA/POE, respectively. C2 shows similar pHRR and THR values as C1, while C3 shows the lowest pHRR and THR of 169 kW/m^2^ and 49.8 MJ/m^2^, respectively. In comparison with the EVA/POE, the pHRR and THR of C3 are decreased by 62% and 42%, respectively. For the total smoke production (TSP), the TSP of EVA/POE/MH is 4.7 m^2^, while that of C3 is only 1.8 m^2^. C1 and C2 show similar TSP values. In comparison with the EVA/POE, the TSPs of the EVA/POE/MH and C3 are decreased by 18% and 68%, respectively.

As seen in Figure 6a, the HRR curve of the EVA/POE/MH has two peaks, while those of the composites from the three processing schemes present only one peak, indicating their obviously suppressed heat release behaviors. Meanwhile, as shown in Figure 6c, the suppressed smoke production behaviors are also revealed in the composites from the three processing schemes. In this research, C3 illustrates lower THR and TSP values than C1 and C2. These results reveal the different actions of the filler distribution structure on the combustion behaviors. In the forced combustion situations of the CONE testing, the segregated structure with the MH-rich center and BN-rich edge in the filled POE phase is favorable to modulation of the combustion behaviors. 

**Figure 7:**
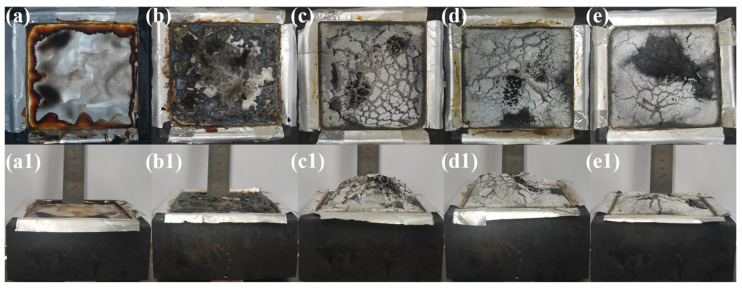
The digital photographs of the residues after CONE testing of POE/EVA (**a**,**a1**), EVA/POE/MH (**b**,**b1**), C1 (**c**,**c1**), C2 (**d**,**d1**) and C3 (**e**,**e1**).

Photographs of the residues after CONE testing in Figure 7 provide further evidence. After testing, the EVA/POE burned almost to the ground without any residues, and the EVA/POE/MH presents powdered residues with fractured and fragmented surfaces. In contrast, the composites from the three processing schemes show the continuously morphological structure of the residues. The residues of C3 are more integrated and compact, with much less fractured surface. Note that swelling phenomena are observed in these composites. Among them, C1 yields the highest residue, while C3 shows the lowest residue. The higher the residue is, the more fractures that are present. There may be two factors for the phenomena, one is the temperature at the combustion surface, the other is the strength of the char surface. A lower surface temperature and stronger char layer favor the formation of char residue for C3.

### 3.6. Char Structure Modulated by Filler Distribution

**Figure 8:**
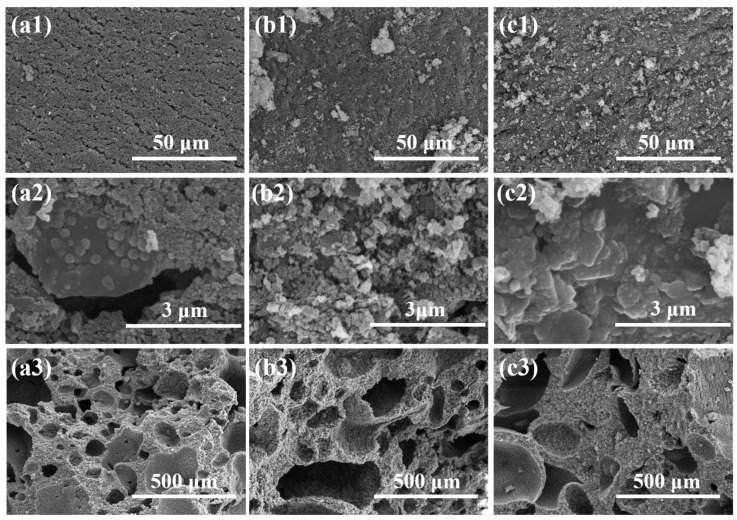
The SEM images of the residual char after CONE testing of C1 (**a1**,**a2**,**a3**), C2 (**b1**,**b2**,**b3**) and C3 (**c1**,**c2**,**c3**), where the low magnified surface structure is designated as 1, the high magnified surface structure is designated as 2 and the char inner structure is designated as 3.

The char structure has been revealed to show strong dependence on the filler distribution, and exerts remarkable influences on the combustion behaviors of the composites. In order to reflect the structural effect, the morphological structure of the residual char after CONE testing of the composites from the three processing schemes is compared in the SEM images of Figure 8.

For C1, the surface char is full of fractured structures, and the inner char is composed of a foam structure with open cells. As a result of the randomly distributed BN and MH in the filled POE phase of C1, the residue components of MgO and BN fail to form a continuous char, and give rise to gaps in the char structure, which can be the main reason for the weak strength of the char and its poor barrier effects. For C2, the surface char is of a continuous structure, and the inner char is composed of a foam structure with less open cells. The segregated structure of the BN-rich center and MH-rich edge in the filled phase makes for the layered char, with MgO residues covering the carbonized structural layer. Similar phenomena are disclosed in the char of C3. The difference between C3 and C2 is that the inner char is mainly composed of a foam structure with much fewer open cells, and a layered char with BN residues covers the structural layer composed of MgO and carbonized residue. The reason for the structured char is due to the segregated structure of the MH-rich center and BN-rich edge in the filled phase of C3.

**Figure 9:**
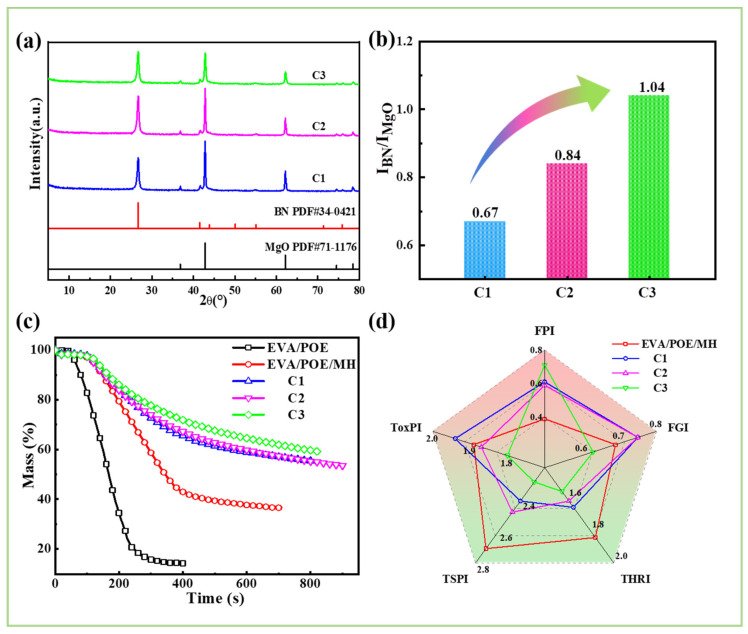
XRD patterns of the residues on the char surface (**a**) and the intensity ratio of the diffraction peak of BN and MgO (**b**); mass curves during CONE testing (**c**) and the radar curves of fire safety parameters of the composites (**d**).

According to the morphological results of the char, the structure of the surface char plays critical roles on the combustion behaviors of the composites. The surface residual char after CONE testing was sampled and characterized via XRD. As shown in Figure 9a, all of the samples show diffraction peaks of MgO (PDF 71-1176) and BN (PDF 34-0421), confirming that the surface char is mainly composed of BN and MgO decomposed from MH. To denote the compositions of the surface char, the relative content ratio of BN and MgO is expressed by the ratio of the diffraction peak intensity of BN at 26.76° to that of MgO at 62.22° (I_BN_/I_MgO_). As shown in Figure 9b, I_BN_/I_MgO_ shows an order of C3 > C2 > C1, indicating that the compositions of the surface char are dependent on the structure of the composites, and C3 shows a higher BN content on the surface char. With more BN on the surface char, a better heat dissipation capacity and barrier effect of the char are expected due to the good thermal conductivity of BN and its small particle size. 

In view of the composition and morphology of the char residue, the filler distribution has an impact on the char performance in two aspects. Firstly, the segregated distribution of BN and MH acts on the formation of the integrated and compact char structure with less fractured surface and densified bulk body. The structured char functions as a barrier to prevent heat transfer and mass loss. As shown in Figure 9c, the distribution structure acts on the mass loss during combustion, showing an ordered loss of C3 < C2 < C1. Secondly, the segregated distribution of BN and MH acts on the formation of BN-enriched and intact layered surface char. Thus, the heat dissipation to the ambience is promoted by the BN-enriched surface, and heat transfer to the condensed phase is prohibited by the intact layered surface. Consequently, the heat and smoke released during CONE testing are significantly suppressed and the fire safety is remarkably enhanced, as shown in Figure 9d.

## 4. Conclusions

BN and MH were adopted as a thermally conductive filler and halogen-free inorganic flame retardant in EVA/POE blends. The selective distribution of BN or MH in the POE phase of the blend was revealed, and the co-continuous network of the composites was built with the formation of the EVA phase and the filled POE phase. When BN and MH were filled together in EVA/POE, three processing schemes were designed to obtain three kinds of the composites with different distributed structures in the filled POE phase, namely, C1 with randomly distributed fillers; C2 with a BN-rich center and MH-rich edge; and C3 with an MH-rich center and BN-rich edge. The segregated distribution of BN and MH was disclosed in C2 and C3. Since BN and MH were found to be located together in the filled POE phase, their selective distribution behaviors were proven to contribute to the formation of the distributed structure.

The thermal conductivity and flame retardancy of the composites show strong dependence on the fillers and their distributions. BN can act more effectively than MH in enhancing the thermal conductivities and heat transfer capabilities of the composites, while MH can act more effectively than BN in enhancing the flame-retardant properties and fire safety performances of the composites. When BN and MH are filled together, the thermal conductivity and flame retardancy of the composites are modulated by the distributed structure of BN and MH. In contrast to the randomly distribution structure of C1, the segregated structure of C2 and C3 proved to be more helpful in enhancing thermal conductivity and flame retardancy. When the contents of BN and MH are both 25 wt.%, C2 shows a thermal conductivity of 0.70 W/(m·K) and an LOI of 27.7%, which is very close to EVA/POE/BN with a thermal conductivity of 0.72 W/(m·K) and EVA/POE/MH with an LOI of 27.6% at the same total filler content of 50 wt.%.

For the combustion behaviors, the composites with the designed distribution structure of BN and MH show a strong impact on heat and smoke release. Significant synergistic effects on suppressing heat release and smoke production were found for the three kinds of the composites with both BN and MH. The char structure was proven to show strong dependence on the filler distribution, and exerted a remarkable influence on the combustion behaviors of the composites. The segregated structure of an MH-rich center and a BN-rich edge in the filled POE phase of C3 was shown to act on the formation of an integrated and compact residue char with improved barrier properties and heat dissipation capacity. C3 shows pHRR, THR and TSP values of 169 kW/m^2^, 49.8 MJ/m^2^ and 1.8 m^2^, which are decreased by 40%, 33% and 62% in comparison with EVA/POE/MH, respectively.

## Data Availability

Data are contained within the article.

## Supplementary Materials

The following supporting information can be downloaded at: https://www.mdpi.com/article/10.3390/polym16050646/s1. Table S1. Compositions of the composites. Table S2 Processing scheme of the EVA/POE/MH/BN compositions. Table S3 The parameters during vertical burning test. Table S4 The combustion parameters of the composites. Figure S1 The EDS spectra at the different positions of C2 (a) and C3 (b).
