# Supplementary material for: Modulating Thermal Conductivity and Flame Retardancy of Polyolefin Composites via Distributed Structures of Magnesium Hydroxide and Hexagonal Boron Nitride
Source: Polymers (Basel). 2024 Feb 28;16(5):646. doi: 10.3390/polym16050646 (PMC10934014; doi:10.3390/polym16050646)
Supplement: Supplementary file 1 [file polymers-16-00646-s001.zip › polymers-2876208-supplementary.pdf]

1. Compositions of the composites

The composites were designed according to the EVA/POE mass ratio of 1/1, the MH/BN mass ratio of 1/1, and the filler content of 50 wt.%. The composites were prepared according to the procedure in the part of 2.2. The samples and their compositions were given in Table S1 and Table S2.

Table S1 Compositions of the composites

Sample	EVA (wt%)	POE (wt%)	MH (wt%)	BN (wt%)
EVA/POE	50	50	-	-
EVA/POE/MH	25	25	50	-
EVA/POE/BN	25	25	-	50
EVA/POE/MH/BN	25	25	25	25
(EVA/MH)/(POE/BN)	25	25	25	25
(EVA/BN)/(POE/MH)	25	25	25	25

Table S2 Processing scheme of the EVA/POE/MH/BN compositions

Sample	Scheme	Processing method
C1	Scheme I	Melt-blending EVA, POE, MH and BN to form EVA/POE/MH/BN
C2	Scheme II	Melt-blending EVA and MH to form (EVA/MH), melt-blending POE and BN to form (POE/BN), melt-blending (EVA/MH) and (POE/BN) to form (EVA/MH)/(POE/BN)
C3	Scheme III	Melt-blending EVA and BN to form (EVA/BN), melt-blending POE and MH to form (POE/MH), melt-blending (EVA/BN) and (POE/MH) to form (EVA/BN)/(POE/MH)

2. The distributed structure of MH and BN

EDS is used to investigate the elemental structure of the composites. Figure S1(a) gives the EDS spectra at the different SEM positions of C2 inserted in Figure 3(d), and Figure S1(b) gives the EDS spectra at the different SEM positions of C3 inserted in Figure 3(e).

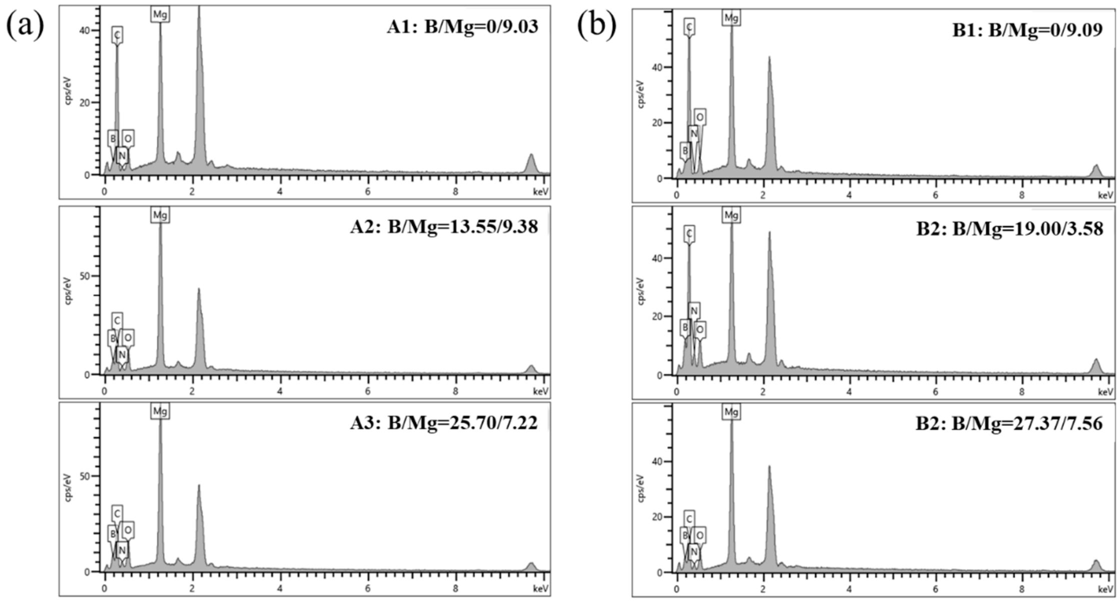

Figure S1 The EDS spectra at the different positions of C2 (a) and C3 (b).

3. Flame retardant properties of the composites

The parameters during the vertical burning test are listed in Table S3.

Table S3 The parameters during vertical burning test

Sample	Burning to the Fixture	Time to burn out (s)	Dripping	Time to dripping (s)	UL 94
EVA/POE/BN	Yes	175	Yes	127	N.R.
EVA/POE/MH	Yes	210	Yes	28	N.R.
C1	Yes	290	Yes	93	N.R.
C2	No	342	Yes	135	N.R.
C3	Yes	289	Yes	90	N.R.

4. Combustion behaviors of the composites

The combustion parameters during the Cone testing are listed in Table S4.

Table S4 The combustion parameters of the composites

Sample	POE/EVA	POE/EVA/MH	C1	C2	C3
TTI (s)	61	111	109	105	120
tpHRR (s)	180	315	130	130	150
pHRR (kW/m^2)	449	281	178	177	169
THR (MJ/m^2)	85.7	74.8	54.7	56.8	49.8
TSP (m^2)	5.7	4.7	2.3	2.7	1.8
FPI (s·m^2/kW)	0.07	0.39	0.61	0.59	0.71
FGI (kW/(m^2·s))	2.97	0.69	0.75	0.75	0.63
$THRI_{6min}$ (MJ/m^2)	1.93	1.84	2.41	2.48	2.29
$TSPI_{6min}$ (m^2)	0.96	1.89	1.94	1.87	1.80
